# Impact of inflammation-mediated response on pan-coronary plaque vulnerability, myocardial viability and ventricular remodeling in the postinfarction period - the VIABILITY study: Protocol for a non-randomized prospective clinical study

**DOI:** 10.1097/MD.0000000000015194

**Published:** 2019-04-26

**Authors:** Mirabela Morariu, Roxana Hodas, Theodora Benedek, Imre Benedek, Diana Opincariu, Andras Mester, Monica Chitu, Istvan Kovacs, Ciprian Rezus, Dan Pasaroiu, Noemi Mitra, Sándor M. Szilágyi, Dan Georgescu, Elena Rezus

**Affiliations:** a Clinic of Cardiology, University of Medicine, Pharmacy, Sciences and Technology of Targu-Mures; b Department of Advanced Research in Multimodality Cardiovascular Imaging, Cardio Med Medical Center, Targu-Mures; c University of Medicine and Pharmacy ‘Gr.T.Popa’, Iasi; d Department of Informatics, Faculty of Science, University of Medicine, Pharmacy; e Department of Internal Medicine, University of Medicine, Pharmacy, Sciences and Technology of Targu-Mures, Romania.

**Keywords:** acute myocardial infarction, inflammation status, myocardial viability, plaque vulnerability, ventricular remodeling

## Abstract

**Introduction:**

While the role of inflammation in acute coronary events is well established, the impact of inflammatory-mediated vulnerability of coronary plaques from the entire coronary tree, on the extension of ventricular remodeling and scaring, has not been clarified yet.

**Materials and methods:**

The present manuscript describes the procedures of the VIABILITY trial, a descriptive prospective single-center cohort study. The main purpose of this trial is to assess the link between systemic inflammation, pan-coronary plaque vulnerability (referring to the plaque vulnerability within the entire coronary tree), myocardial viability and ventricular remodeling in patients who had suffered a recent ST-segment elevation acute myocardial infarction (STEMI). One hundred patients with STEMI who underwent successful revascularization of the culprit lesion in the first 12 hours after the onset of symptoms will be enrolled in the study. The level of systemic inflammation will be evaluated based on the serum biomarker levels (hs-CRP, matrix metalloproteinases, interleukin-6) in the acute phase of the myocardial infarction (MI) and at 1 month. Pan-coronary plaque vulnerability will be assessed based on serum biomarkers known to be associated with increased plaque vulnerability (V-CAM or I-CAM) and at 1 month after infarction, based on computed tomographic angiography analysis of vulnerability features of all coronary plaques. Myocardial viability and remodeling will be assessed based on 3D speckle tracking echocardiography associated with dobutamine infusion and LGE-CMR associated with post-processing imaging methods. The study population will be categorized in 2 subgroups: subgroup 1 - subjects with STEMI and increased inflammatory response at 7 days after the acute event (hs-CRP ≥ 3 mg/dl), and subgroup 2 - subjects with STEMI and no increased inflammatory response at 7 days (hs-CRP < 3 mg/dl). Study outcomes will consist in the rate of post-infarction heart failure development and the major adverse events (MACE) rate.

**Conclusion:**

VIABILITY is the first prospective study designed to evaluate the influence of infarct-related inflammatory response on several major determinants of post-infarction outcomes, such as coronary plaque vulnerability, myocardial viability, and ventricular remodeling.

## 1 Introduction

### 1.1 Background and rationale

It is well-known that the highest risk of death following an acute myocardial infarction (AMI) is within the first hours from onset. In the latest years, introduction of coronary care units and large-scale implementation of networks for emergency myocardial revascularization, as well as development of reperfusion and pharmacological therapies, led to a significant decrease in mortality rates following AMI. However, the high incidence of post-infarction heart failure is still affecting the long-term outcomes of these patients.^[1–4]^

Congestive heart failure (CHF) as a long-term complication following AMI, although having a relatively low incidence in the primary percutaneous coronary intervention (PCI) era, is an important predictor of mortality and morbidity, at the same time leading to increased health-care costs and decreased quality of life.^[5]^ Predictors for CHF following an AMI include preexistent heart failure, traditional cardiovascular risk factors (diabetes, hypertension, old age, smoking, chronic renal disease), previous myocardial infarction (MI) with or without revascularization, rhythm disturbances during the acute phase of the coronary event, delayed revascularization times, multivessel coronary artery disease (CAD), enhanced inflammatory response, or no-reflow phenomenon after revascularization.^[6–9]^

Multiple coinciding pathophysiological mechanisms may lead to heart failure after AMI, both during the index event and later on. These include myocardial necrosis and stunning, inflammation, reperfusion injury, microvascular obstruction, fibrosis and remodeling. CHF during the acute phase of an acute coronary syndrome results from a combination of myocardial necrosis and stunning, as well as ischemic (including papillary muscle dysfunction and subsequent mitral regurgitation, cardiac tamponade, ventricular overload) and arrhythmic complications. Later onset of CHF arises as a consequence of myocardial scarring and remodeling.^[5,10–12]^

The major aim of the urgent revascularization in ST-elevation myocardial infarction (STEMI) is to save myocardial tissue from death and necrosis, in order to recover viable myocardium. Despite a timely revascularization of the epicardial culprit coronary artery, a significant proportion of patients do not present the typical signs for a successful reperfusion, this being defined as the no-reflow phenomenon. Various mechanisms have been incriminated in an attempt to provide an explanation for this phenomenon, including microvascular embolization, endothelial dysfunction due to an exaggerated inflammatory response, or reperfusion injury. The consequences of this phenomenon are expansion of the infarct size, life-threatening arrhythmias, early development of CHF, and enhanced ventricular remodeling and dilation. Diagnosis of no-reflow can be achieved in the catheterization laboratory, during the revascularization procedure, or via imaging techniques such as gadolinium contrast magnetic resonance, contrast echocardiography or nuclear imaging.^[13–16]^

It is well-known that one of the major factors that trigger an AMI is the systemic inflammation, which has a direct influence on coronary plaque vulnerability.^[17]^ Atheromatous plaques develop and progress due to a chronic non-resolving inflammatory process that occurs at the site of an endothelial injury caused by turbulent blood flow. The atherosclerotic lesions can evolve in a silent manner for a number of years without causing an acute event.^[18]^ However, some plaques become unstable by acquiring several phenotypic changes (large necrotic core, thin cap fibroatheroma, microcalcifications, vascular remodeling) which make them prone to rupture.^[19,20]^ Several studies have shown that vulnerable plaques are associated with an enhanced inflammatory response, which causes overexpression of the vulnerability features, with consequent rupture and thrombosis.^[20–22]^ Furthermore, myocardial injury triggers an inflammatory cascade, which is persistent during the first 3 to 5 days following the acute event, having a benefic reparatory effect.^[23]^ The persistence of an elevated systemic inflammation after AMI after day 3 to 5 has deleterious effects, stimulating the process of left ventricular remodeling and evolution towards CHF. Moreover, the enhanced inflammation secondary to myocardial ischemia, may lead to vulnerabilization of additional atherosclerotic plaques thorough the vascular system, which might explain increased rates of acute coronary and cerebrovascular events in patients that have already suffered an AMI.^[8,24,25]^

The exact influence of various pathophysiological mechanisms, such as no-reflow phenomenon or increased inflammatory response on a pan-coronary level, on the process of ventricular remodeling is not fully understood. Understanding the complex heterogenous interaction of these components could help to identify patients at a higher risk for developing heart failure, as well as subsequent coronary and cerebrovascular events, after they have already suffered an AMI.

The present manuscript describes the procedures of the VIABILITY trial, a descriptive prospective single-center cohort study which intends the assessment of the link between systemic inflammation, pan-coronary plaque vulnerability (referring to the plaque vulnerability within the entire coronary tree), myocardial viability and ventricular remodeling in patients who had suffered a recent STEMI.

### 1.2 Study objectives

The *primary objective* consists in the evaluation of the relationship between coronary inflammation-mediated plaque vulnerability, myocardial viability and ventricular remodeling in the post-infarction period.

The *secondary objectives* of the study are:

(1) to assess the effect of systemic inflammation on the structural remodeling process of the left ventricle (LV) following an AMI and
(2) to evaluate the impact of local inflammatory response on pan-coronary plaque vulnerability in the post-infarction period.

## 2 Methods/design

### 2.1 Study design

VIABILITY is a prospective, non-randomized, cohort study, carried out in a single-center, which aims to assess the link between systemic inflammation, pan-coronary plaque vulnerability (referring to the plaque vulnerability within the entire coronary tree), myocardial viability and ventricular remodeling in patients with recent STEMI.

### 2.2 Ethics approval

The VIABILITY clinical study is approved by the local Ethics Committee for Scientific Research of the University of Medicine and Pharmacy of Tirgu-Mures, Romania (certificate of approval: 349/13.12.2017) and the Ethics Committee for Scientific Research of the Cardio Med Medical Center Tirgu Mures, Romania (certificate of approval: 30/28.12.2017). All study procedures will be conducted according to the *Declaration of Helsinki*. Each subject will provide sign written informed consent before randomization process.

### 2.3 Study population

VIABILITY will be a single-center, observational, non-randomized study including 100 patients with STEMI who underwent successful revascularization of the culprit lesion in the first 12 hours after the onset of symptoms.

#### 2.3.1 Inclusion criteria

- Patients with STEMI treated by primary PCI within the first 12 hours after the onset of symptoms;
- Body mass index (BMI) under 40 kg/m^2^;
- Signed written informed consent;
- Subject at age of majority.

#### 2.3.2 Exclusion criteria

- Documented ACS within last month before randomization;
- Patient disapproval to provide written informed consent;
- Intolerance to iodine contrast agent;
- Conditions associated with contraindication for CMR examination;
- Women during pregnancy or lactation period;
- Women able to procreate without any contraceptive usage;
- Chronic kidney disease (glomerular filtration rate <60 ml/min/1.73 m^2^) or acute renal injury that requires hemodialysis;
- Any type of neoplasia documented in the last 3 years before randomization;
- Expectation of life <1 year.

### 2.4 Study settings

The VIABILITY study will be carried out in the Center of Advanced Research in Multimodality Cardiac Imaging of the Cardio Med Medical Center and in the Clinic of Cardiology of the County Clinical Emergency Hospital Tirgu Mures, affiliated to the University of Medicine, Pharmacy, Sciences and Technology of Tirgu Mures, Romania. Funding will be provided via research grant number UEFISCDI PN-III-P2–2.1 BG-2016–0343 – COROFLOW (contract 43/05.09.2016, full name: New technologies for 3D computational simulation of coronary circulation and myocardial perfusion based on fusion imaging), financed by the European Union and the Romanian Government through the Ministry of Education and Research, which was selected for funding in a national competition for research grants, following an international peer-review selection process.

### 2.5 Study population and subgroups

This study will enroll 100 patients with STEMI meeting the inclusion criteria. Study subjects will be distributed in 2 groups: group 1 – patients with STEMI and increased inflammatory response at 7 days after the acute event (hs-CRP ≥ 3 mg/dl) and group 2 - subjects with STEMI and no increased inflammatory response at 7 days (hs-CRP <3 mg/dl).

### 2.6 Study procedures and outcome assessment

- Medical records, physical exam, laboratory analysis: complete blood count (CBC), biochemistry, levels of high sensitivity C-reactive protein (hs-CRP), MMPs, IL6, NT-pro-BNP, adhesion molecules;
- electrocardiogram (ECG);
- TTE with morphological measurements, assessment left ventricular systolic and diastolic performance, speckle tracking echo, Dobutamine viability test;
- LGE-CMR with the evaluation of myocardial scar and fibrosis, ventricular function and remodeling index;
- Angiographic Computed Tomography (Angio-CT) with assessment of coronary plaque vulnerability (based on vulnerability features: positive remodeling, spotty calcification, napkin-ring sign, low-density plaque) and myocardial perfusion.

#### 2.6.1 Biomarkers assays

Routine biochemistry and CBC analysis will be performed at the main laboratory of the Emergency Clinical County Hospital of Tirgu-Mures, immediately after randomization. Inflammatory status (hs-CRP levels) will be evaluated at 7 days after the acute cardiac event via immunoturbidimetric assay. Adhesion molecules (VCAM, ICAM), matrix metalloproteinase 9, and interleukin-6 serum levels will be evaluated using enzyme linked immunosorbent assay. Serum inflammatory biomarkers will be analyzed in Advanced Medical and Pharmaceutical Research Center of the University of Medicine and Pharmacy Tirgu-Mures, Romania. N-terminal prohormone of brain natriuretic peptide (NT-proBNP) levels will be evaluated at randomization and at the last follow-up visit, at the main laboratory of the Emergency Clinical County Hospital of Tirgu-Mures by electrogenerated chemiluminescence.

#### 2.6.2 2D transthoracic echocardiography

Transthoracic echocardiographic (TTE) assessment will be performed at first and last follow-up visit in all patients. Evaluation of cardiac diameters and volumes, systolic and diastolic performance of LV, speckle tracking, and Dobutamine viability test will be conducted with a Vivid E9 ultrasound system (General Electric Vingmed Ultrasound, Horten, Norway), with the patient turned slightly on his left side, according to American College of Echocardiography indications. Left ventricular end-systolic volume (LVESV), left ventricular end-diastolic volume (LVEDV), and left ventricle ejection fraction (LVEF) will be evaluated using the Simpson's method, on the basis of images obtained from apical four chamber view. All measurements will be calculated as an average from 3 heart cycles.

#### 2.6.3 Late gadolinium-enhancement cardiac magnetic resonance (LGE-CMR)

All cardiac magnetic resonance images will be acquired with the use of a 1.5T Siemens Magnetom Aera equipment. The structure and function of the LV will be assessed by cine acquisitions in the long axis, 2-chamber and 4-chamber view respectively, as well as short axis, from the mitral plain through the apex. Short axis cine images will also be used for assessment of LV mass, LVEF, end-diastolic and end-systolic volumes of the LV. The LGE-CMR will be used to evaluate the myocardial scar, degree of fibrosis and the remodeling process of the LV. The images will be acquired during the diastolic phase of the cardiac cycle, by using the same views as for cine imaging, respectively short and long axis views, but after a 10 minutes delay after gadolinium intravenous injection. Imaging data post-processing will be conducted with the use of Medis Q Mass 8.1 software (Medis, Leiden, the Netherlands), by manually tracing the epicardium and endocardium. The analysis of the delayed signal intensity (DSI) will be conducted in 10 to 12 successive short-axis LGE slices. Image segmentation will use an automated hyper-enhancement threshold, and myocardial regions with a slow washout kinetic will be considered as fibrotic areas. The volume of the epicardial adipose tissue will be measured by a semiautomatic protocol for image post-processing, using automatic tracing and manual adjustments for epicardial layers.

#### 2.6.4 Coronary computed tomographic angiography

Coronary Computed Tomographic Angiography associated with complex post-processing will be used for assessment of pan-coronary plaque vulnerability (based on vulnerability features: positive remodeling, spotty calcification, napkin-ring sign, low-density plaque), myocardial perfusion and for quantification of epicardial fat volume, using a 128-slices dual-source computer tomography (CT) scanner (SOMATON Definition, Siemens Healthcare, Erlangen, Germany) and the work station Siemens Syngo Via Frontier – Coronary Plaque Analysis platform (Siemens Medical Solutions, Erlangen, Germany), available in the Laboratory of Advanced Research in Cardiac Multimodal Imaging of the Cardio Med Medical Center, Targu Mures, Romania.

### 2.7 Study timeline

VIABILITY study will be conducted from March 2019 to March 2021.

### 2.8 Outcomes

The *primary outcome* of the study refers to the incidence of post-infarction heart failure development, defined as the rate of re-admission in the hospital for heart failure or by a significant decrease in the ejection fraction (<45%).

*Secondary outcomes* of the study are represented by rate of re-hospitalization rates, rate of repeated revascularization and major adverse events (MACE) rates, including cardiovascular death or stroke.

### 2.9 Participation timeline

#### 2.9.1 Baseline (day 0)

- Achieve written informed consent form all patients.
- Check all inclusion/exclusion criteria.
- Record demographic information, medical records, cardiovascular risk factors.
- Perform and record physical examination and 12-lead ECG.
- Laboratory analysis (CBC, routine biochemistry, inflammatory biomarkers, acute adhesion molecules).
- 2D TTE/speckle tracking.

#### 2.9.2 Visit 1 (day 7 / discharge from the hospital)

- hs-CRP assessment

#### 2.9.3 Visit 2 (month 1)

- Angio-CT (coronary plaque vulnerability features assessment, epicardial fat, myocardial perfusion).
- LGE-CMR (myocardial fibrosis/scar, remodeling, viability).
- Dobutamine stress echocardiography/speckle tracking (viability).

#### 2.9.4 Visit 3,4,5 (month 3,6,9)

- Record results of physical exam, medical records, ECG
- 2D TTE

#### 2.9.5 Final study visit (month 12)

- Record results of physical exam, medical records, ECG
- 2D TTE
- End-point assessment

#### 2.9.6 Study procedures

- Medical records, physical exam, laboratory analysis (CBC, biochemistry, serum levels of hs-CRP, MMPs, IL6, NT-pro-BNP, adhesion molecules);
- ECG.
- TTE with morphological measurements, assessment left ventricular systolic and diastolic performance, speckle tracking echo, Dobutamine viability test.
- LGE-CMR with the evaluation of myocardial scar and fibrosis, ventricular function and remodeling index.
- Angio-CT with assessment of coronary plaque vulnerability (based on vulnerability features: positive remodeling, spotty calcification, napkin-ring sign, low-density plaque) and myocardial perfusion.

**Figure 1:**
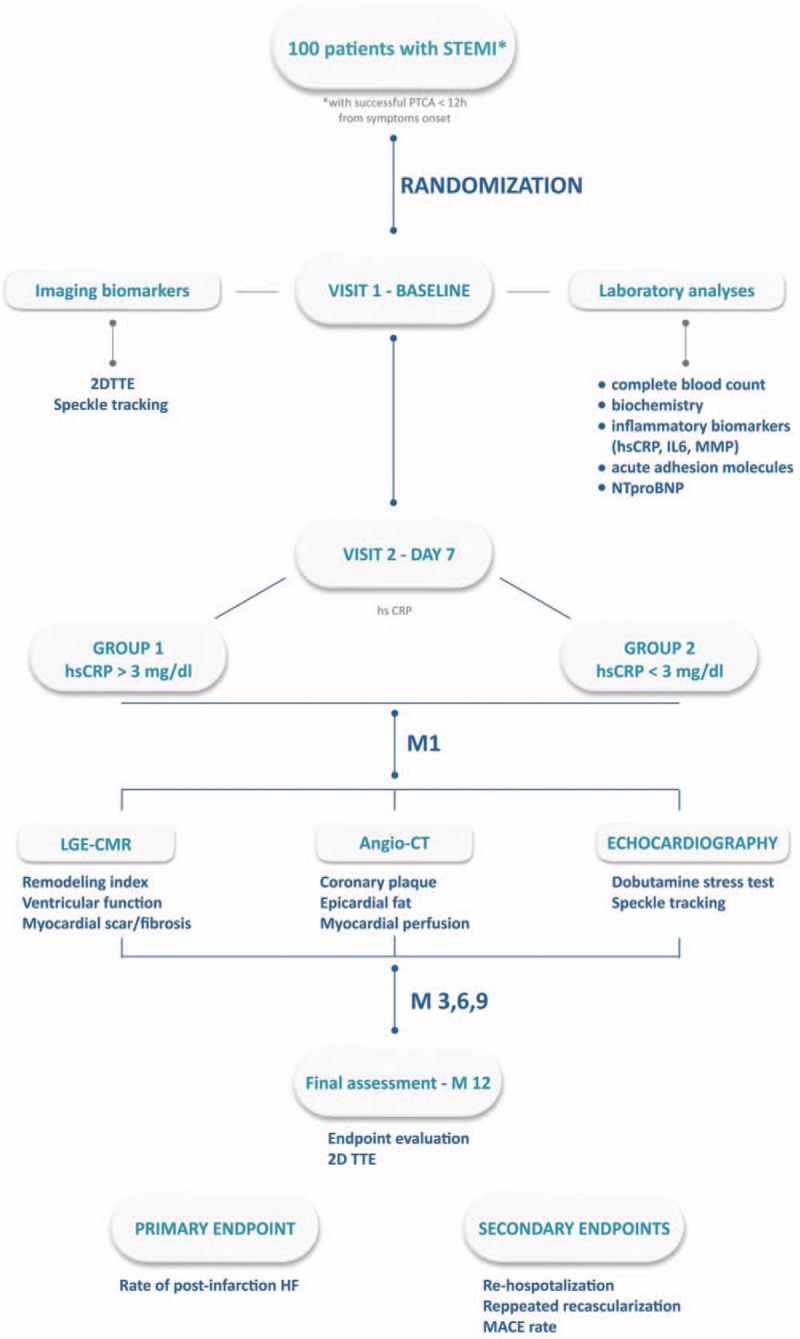
Study flowchart.

All study procedures are illustrated in Figure 1, which presents the VIABILITY flowchart.

### 2.10 Sample size

One hundred patients with STEMI who underwent successful revascularization of the culprit lesion within the first 12 hours after the onset of symptoms will be enrolled in this study. On the basis of the persistence of systemic inflammation at 7 days after the acute event patients will be allocated into 2 subgroups. Stat Mate 2.0 software was used in order to establish the sample size of the study. According to this computation, a sample size of 50 subjects per group presents a 90% power to detect an increase in major adverse CV events rates proportion of 3.28, with a significance level (alpha) of 0.05 (2-tailed). Therefore, the study population was established at 100 subjects.

### 2.11 Statistical analysis

Graph Pad in Stat 3.10 software (GraphPad Software, San Diego, CA) will be used in order to perform all the statistical analysis, using a level of significance of 5%. Normality tests will be performed in order to check all study data. Categorical variables will be interpreted with Pearson Chi-Square test and being presented as numerical or percentage values respectively. Continuous variables will be presented in the form of a mean value +/- SD and will be compared using the Student test for variables with gaussian distribution and Mann–Whitney test for variable with non-gaussian distribution.

## 3 Discussions

This manuscript presents the protocol of a clinical study, conducted in a single center, with the purpose to analyze the link between systemic inflammation, pan-coronary plaque vulnerability, myocardial viability and remodeling in patients who had suffered a recent STEMI.

It is well-known that one of the major factors that trigger an AMI is the systemic inflammation, which has a direct influence on coronary plaque vulnerability, usually related to decreased structural stability.^[26]^ According to histological studies, prone-to-rupture plaques present significant differences as compared with stable obstructive coronary plaques.^[27]^ Those high-risk plaque features associated with the presence of acute coronary syndrome (ACS) have been defined by a series of Angio-CT studies and include larger plaque volume, plaque with low attenuation, positive remodeling, large plaque burden, spotty calcium, and napkin-ring sign. Ruptured plaques, the main cause of MIs, proved to have fibrous caps that contain elevated levels of proteases, including metalloproteinases (MMPs). Histologic studies provide strong associative evidence that MMPs promote plaque vulnerability in humans.^[28]^ Similarly, two overexpression studies give a clear evidence of plaque disruption in the presence of high MMP-9 levels, that are thought to influence the rates of atherogenesis and the stability of atherosclerotic plaques.^[29,30]^

Therefore, one of the study objectives will be to investigate the impact of local inflammatory response, assessed by inflammatory biomarkers and adhesion molecules, on pan-coronary plaque vulnerability based on vulnerability features determined via coronary CT angiography, following an AMI.

Patients with AMI who survive the acute event very often present significant deterioration of their clinical status with progression towards heart failure, which is directly linked to the extent of myocardial injury and the remodeling process starting immediately after infarction. At the same time, 20% of patients surviving an AMI present a recurrent cardiovascular event in the following 12 months, as a result of increased systemic inflammation triggered by the infarction, which leads to vulnerabilisation of other atheromatous plaques from all the circulatory system in the post-infarction period. While the role of inflammation in acute coronary events is well established, the impact of inflammatory-mediated vulnerability of coronary plaques from the entire coronary tree on the extension of ventricular remodeling and scaring has not been elucidated so far.

Inflammatory response triggered by MI leads to appropriate infarct healing process and cardiac repair. Despite its beneficial effect, inflammatory reaction present in post AMI phase is a double-edged sword that in association with a vulnerable plaque, vulnerable blood and vulnerable myocardium can lead to catastrophic events, causing postinfarction remodeling and heart failure.^[26,31]^ Based on multivariate analyses, a series of previous studies reported a severe inflammatory response, expressed by serum CRP levels >20 mg/dl, as an independent strong predictor of subacute cardiac rupture and ventricular aneurysm.^[31]^ Furthermore, a prospective study has demonstrated an association between inflammation and LV remodeling, as the group of patients with peak CRP level above the median had a greater increase in LV volumes both at 2 weeks and at 6 months.^[31]^

In recent studies, CMR parameters, including infarct size assessed by LGE, have been extensively evaluated in predicting late LV function in patients with AMI undergoing primary percutaneous intervention (PCI), providing powerful surrogate markers of outcomes.^[32]^ At a clinical level, infarct size proved to be an independent prognostic factor for heart failure, arrhythmic events, and cardiac mortality.^[33]^ In PROTECTION AMI CMR study, the infarct size evaluated by LGE-CMR on day 3 to 5 proved to be the most robust independent predictor of late LVEF and adverse LV remodeling, at 90 days post STEMI.^[34]^ Negative remodeling, based on the cut-off value of >20% left ventricle end-diastolic (LVED) dilatation at 6 months follow-up, proved to have prognostic importance in 2 large multicenter trials.^[35]^ Moreover, in a study conducted by Haeck et al, NT-proBNP proved to be the strongest independent predictor of LV function, assessed by CMR imaging, in patients with STEMI undergoing PCI.^[36]^ As smaller previous studies have reported, admission levels of NT-proBNP above 260 pg/ml proved to be in a strong correlation with LV remodeling and impaired cardiac function during long-term follow-up.^[3]^

## 4 Conclusions

The primary contribution of the VIABILITY trial will be to elucidate the consequence of infarct-related inflammatory response on several major determinants for post-infarction outcomes, such as coronary plaque vulnerability, myocardial viability, and ventricular remodeling.

## Author contributions

Submission to ethical committee was performed by Mirabela Morariu. The radiologist responsible for CMR imaging interpretation is Mihaela Ratiu. Morariu Mirabela, Sándor M. Szilágyi and Theodora Benedek will perform data statistical analysis.

**Conceptualization:** Morariu Mirabela, Diana Opincariu, Roxana Hodas, Ciprian Rezus, Andras Mester, Istvan Kovacs, Szilágyi László, Imre Benedek.

**Data curation:** Theodora Benedek, Kovacs Istvan.

**Formal analysis:** Andras Mester, Theodora Benedek.

**Investigation:** Theodora Benedek, Monica Chitu.

**Methodology:** Mirabela Morariu, Opincariu Diana, Elena Rezus, Imre Benedek, Theodora Benedek.

**Resources:** Imre Benedek.

**Supervision:** Imre Benedek, Theodora Benedek.

**Visualization:** Andras Mester.

**Writing – original draft:** Morariu Mirabela, Diana Opicariu, Roxana Hodas, Dan Pasaroiu, Noemi Mitra, Andras Mester, Dan Georgescu, Imre Benedek.

All the authors approved the final manuscript.
